# Disrupted interhemispheric coordination of sensory-motor networks and insula in major depressive disorder

**DOI:** 10.3389/fnins.2023.1135337

**Published:** 2023-03-07

**Authors:** Chunguo Zhang, Huan Jing, Haohao Yan, Xiaoling Li, Jiaquan Liang, Qinqin Zhang, Wenting Liang, Yangpan Ou, Can Peng, Yang Yu, Weibin Wu, Guojun Xie, Wenbin Guo

**Affiliations:** ^1^Department of Psychiatry, The Third People’s Hospital of Foshan, Foshan, Guangdong, China; ^2^Department of Psychiatry, National Clinical Research Center for Mental Disorders, The Second Xiangya Hospital of Central South University, Changsha, Hunan, China

**Keywords:** major depressive disorder, voxel-mirrored homotopic connectivity, support vector machine, magnetic resonance imaging, brain

## Abstract

**Objective:**

Prior researches have identified distinct differences in neuroimaging characteristics between healthy controls (HCs) and patients with major depressive disorder (MDD). However, the correlations between homotopic connectivity and clinical characteristics in patients with MDD have yet to be fully understood. The present study aimed to investigate common and unique patterns of homotopic connectivity and their relationships with clinical characteristics in patients with MDD.

**Methods:**

We recruited 42 patients diagnosed with MDD and 42 HCs. We collected a range of clinical variables, as well as exploratory eye movement (EEM), event-related potentials (ERPs) and resting-state functional magnetic resonance imaging (rs-fMRI) data. The data were analyzed using correlation analysis, support vector machine (SVM), and voxel-mirrored homotopic connectivity (VMHC).

**Results:**

Compared with HCs, patients with MDD showed decreased VMHC in the insula, and increased VMHC in the cerebellum 8/vermis 8/vermis 9 and superior/middle occipital gyrus. SVM analysis using VMHC values in the cerebellum 8/vermis 8/vermis 9 and insula, or VMHC values in the superior/middle occipital gyrus and insula as inputs can distinguish HCs and patients with MDD with high accuracy, sensitivity, and specificity.

**Conclusion:**

The study demonstrated that decreased VMHC in the insula and increased VMHC values in the sensory-motor networks may be a distinctive neurobiological feature for patients with MDD, which could potentially serve as imaging markers to discriminate HCs and patients with MDD.

## 1. Introduction

Major depressive disorder (MDD), a prevalent mental illness, is the foremost cause of disability worldwide (CONVERGE consortium, 2015; Yang et al., 2020). Furthermore, it is a complicated emotional disorder characterized by abnormal clinical symptoms, including cognitive disorders (such as feelings of excessive guilt or worthlessness), autonomic dysfunction (such as changes in appetite or sleep patterns), abnormal psychomotor activities (such as excitement or retardation), and elevated risk of suicide (Lou et al., 2019). Approximately 12% of the global population is affected by MDD, while the lifetime occurrence of MDD in the United States surpasses 20% (Hasin et al., 2018; Hayley et al., 2021; Fries et al., 2022). The incidence of MDD has risen since the outbreak of COVID-19 (COVID-19 Mental Disorders Collaborators, 2021). Approximately 25–50% of those with MDD exhibit deficiencies in one or more cognitive domains, which are considered key features of the condition (Liu et al., 2022).

Researches have demonstrated that MDD is a heritable and heterogeneous disorder (Andersson et al., 2019; Kendall et al., 2021; Nguyen et al., 2022), and its clinical features are associated with brain regions that exhibit anatomical differences. Multiple neuroimaging studies have attempted to identify the neurobiology of MDD and distinguished several neuroimaging characteristics between healthy controls (HCs) and patients with MDD. Zhang et al. (2022) found that, in comparison to HCs, patients with MDD exhibited decreased functional connectivity (FC) between the emotional subregion of the anterior cingulate cortex (ACC) and the hippocampus, thalamus, insula, angular gyrus, and posterior cingulate cortex. On the other hand, other studies reported increased connectivity between the hippocampus and the ACC and reduced connectivity between the insula and the ACC in patients with MDD (Krug et al., 2022). Functional brain imaging studies revealed significant changes in the FC in the cerebellum-neocortex and cerebellum-basal ganglia circuits in patients with MDD (Dai et al., 2022). Additionally, an increased long-range positive FC (lpFC) in the left inferior parietal lobule was found to distinguish HCs and patients with MDD (Dziedzic et al., 2022). Given that the architecture of the connectome affects the effectiveness and velocity of information transport, including regions such as anterior and middle cingulate cortex, medial occipital areas, superior frontal areas, post- and precentral gyrus, parahippocampal gyrus, and precuneus, the balance of intra-hemispheric and inter-hemispheric connectivity plays a crucial role in brain function (Krupnik et al., 2021).

Studies have demonstrated that communication between the left and right hemispheres of the human brain is a crucial aspect of both cognitive and emotional processing (Compton et al., 2005; Toro et al., 2008). Ran et al. (2020) showed that anatomical abnormalities in the corpus callosum in patients with MDD may result from caused by imbalanced inter-hemispheric communication. Concurrently, a magnetic resonance imaging (MRI) study indicated that the deterioration of inter-hemispheric FC is related to the severity of clinical depression and treatment outcomes of patients with MDD (Kozel et al., 2011; Zheng et al., 2022). Additionally, patients with MDD had notably higher overall average (static) functional connectivity (sFC), but lower variability of functional connectivity (vFC) within networks (Demirtas et al., 2016). As per the research by Liu et al. (2021) inter-hemispheric homotopic connection in particular regions could be serve as a potential biomarker to distinguish patients with MDD from HCs. Reduced inter-hemispheric coordination in the posterior default-mode network and visual regions was also revealed between HCs and patients with MDD (Guo et al., 2018).

Voxel-mirrored homotopic connectivity (VMHC) is a technique used to calculate resting-state FC between voxels in a hemisphere and their corresponding mirror regions in the opposite hemisphere (Jia et al., 2020; Fan et al., 2022). VMHC can be utilized to determine the intensity of functional connections between brain regions in both hemispheres that are located at the same position (Zuo et al., 2010; Jin et al., 2021). Several studies have concluded that the mechanism behind VMHC deficiency may be linked to extensive abnormalities in white matter integrity, dysfunction in local gray matter structure, and the mode of pathway reorganization (Yuan et al., 2012; Ding et al., 2015). Moreover, this method can be used to evaluate the relationship between time series dependent on blood oxygen levels, and to demonstrate how information is exchanged between the two hemispheres of the brain (Wei et al., 2018). Some clinical studies have demonstrated altered homotopic FC by measuring VMHC in patients with MDD (Lai and Wu, 2014; Hou et al., 2016). Support vector machine (SVM) is a specialized form of supervised machine learning that multivariable pattern recognition technology that is applied to predict psychosis based on neuroanatomical indicators (Shan et al., 2021). SVM can effectively identify a set of information and features from different brain regions, which can be used to classify patients and HC using neuroimaging data [such as functional magnetic resonance imaging (fMRI) data] (Steardo et al., 2020), making the classification results more convincing. Numerous studies have employed VMHC and SVM methods to study brain disorders, providing possible evidence for the discovery of biological markers in neuroimaging (Chen et al., 2021; Chu et al., 2022; Wu et al., 2022). However, it is still unclear whether abnormal VMHC can be used as an underlying brain imaging symbol to discriminate HCs from MDD using the SVM analysis.

In this study, our objective was to apply the VMHC method to identify the inter-hemispheric functional interaction during resting state in individuals with MDD. This study investigated whether abnormal VMHC might be utilized as a potential marker in patients with MDD by combining VMHC values with cognitive tests, exploratory eye movement (EEM), event-related potentials (ERPs), and other markers. We hypothesized that patients with MDD would exhibit reduced VMHC, which could serve as a potential imaging marker to differentiate between HCs and patients with MDD. In addition, we proposed that aberrant VMHC would be associated with clinical variables in MDD.

## 2. Materials and methods

### 2.1. Participants

Patients with MDD were recruited from Foshan Third People’s Hospital. This study included 32 first-episode patients and 10 recurrent patients with MDD. Forty-two HCs matched for sex and years of education were recruited from the community. The Diagnostic and Statistical Manual of Mental Disorders-5 (DSM-5) patient version is used to determine the diagnosis of MDD. For recurrent patients, the use of antidepressants was suspended for at least 2 weeks by themselves. The anxiety and depressive symptoms, social function, personality characteristics, coping style, social support, and psychological cognitive function of the subjects were measured by Hamilton Anxiety Scale (HAMA), Hamilton Depression Scale (HAMD), Social Disability Screening Schedule (SDSS), Eysenck Personality Questionnaire (EPQ), Simplified Coping Style Questionnaire (SCSQ), Social Support Scale (SSS), Wisconsin Card Sorting Test (WCST), and Repeatable Battery for the Assessment of Neuropsychological Status (RBANS). All participants were right-handed and ranged in age from 18 to 60 years old. The exclusion standards for all subjects were as follows: (1) a history of severe physical sickness or alcohol or drug abuse; (2) significant physical impairment, preventing completion of the follow-up study; and (3) the presence of mental retardation, dementia, and severe cognitive impairment in addition to other serious psychiatric problems.

The study was approved by the Research Ethics Committee of the Third People’s Hospital of Foshan, and a written informed consent was obtained from all participants.

### 2.2. Event-related potentials (ERP) data acquisition

Event-related potential data were collected using a Japanese Kohden MEB-9402C myoelectric evoked potentiometer. Participants were instructed to sit comfortably and make an effort to concentrate. The electrode placement followed the 10/20 standard by the International Electroencephalogram Association. The ground was put in the center of the hand FPz. The right ear M2 point served as the recording electrode. The central Cz point served as the reference electrode. The electrode impedance was set to 5K, and the filter was 0.2–20 Hz. The analysis duration was 1000 ms. The stimulation is carried out in the traditional “Oddball” auditory stimulation mode, with a frequency of 1 times/s, a duration of 10 ms, and a sensitivity of 5 μV. The detection was accomplished by triggering and activating two systems, which were band-pass filtered 200 times with the low-frequency filter and the high-frequency filters. The settings for the non-target stimulus were set to 80% probability, 70 dB intensity, and 1000 Hz frequency, while the parameters for the target stimulus were set to 20% probability, 90 dB intensity, and 2000 Hz frequency. The two frequencies were randomly interspersed, and each scenario was repeated twice before the average value was calculated. The subject was presented with a target stimulus and the non-target stimulus was not utilized as a response. If a test subject had less than 80% hits, the test was considered invalid. The latencies of the N100, P200, N200, and P300 waves were recorded separately.

### 2.3. Exploratory eye movement (EEM) data acquisition

The EEM data was collected using a Shanghai-made Dekang DEM-2000 eye movement detector. The participants were instructed to sit comfortably in a chair and focus on a small screen in front of them. The angle of eyes movement from the left to the right side of the screen was 33°, and the distance between their eyes and the screen was 25 cm. The participants were instructed to pay close attention as the initial S-shaped pattern (S) was displayed on the screen for 15 s. Within this time, the device automatically captured the gaze positions and recorded the number of eye fixations (NEF). After that, the second and third S-shaped patterns (S2, S3), which were slightly different from the first image, were displayed on the screen. Every pattern lasted 15 s. When prompted with the question, “What is the difference between the two patterns,” the participants were instructed to pay close attention before responding with, “There is no difference.” The responsive seeking score was calculated from the gaze points in seven locations (only one point was considered in each region) for a period of 5 s (RSS). The device can automatically record the eye movement’s trajectory, the data are automatically processed by the computer, and the entire procedure may be replayed for later use. A gaze point in EEM analysis is defined as an eye’s gaze time that exceeds 200 ms on specific spot (the eyeball is moving within 2°). This is defined as the total number of gaze points in 15 s when the eye fixes on the S-pattern. The RSS score is broken down into seven S2 or S3 regions, and the instrument counts the NEF areas for a total of 5 s. No matter how often, the subject’s attention on a certain place is worth one point. The maximum RSS score for each picture is thus 7, and for S2 and S3, the maximum RSS overall score is 14. NEF 30 and/or RSS 4 were used as the criterion for abnormality.

### 2.4. Measures

Hamilton Depression Scale (17 items) was used to assess depressive severity. A higher score indicates a more severe level of depressive symptoms (Bagby et al., 2004). The level of anxiety symptoms was evaluated using the HAMA. This scale consists of 14 items, each with a score ranging from 0 to 4. A higher score indicates a greater severity of anxiety symptoms. The SDSS is a 10-item scale that was clinician-administered to assess the level of functional impairment. A three-point Likert scale (0–2) was used to rate each item. A higher score indicates a greater level of functional impairment (Yan et al., 2022). Factor analysis of the EPQ scale revealed three orthogonal dimensions, leading to the proposed of four basic factors to determine personality: Extraversion (E), Neuroticism (N), Psychoticism (P), and Lie (L). These three dimensions reflect individuals’ varying tendencies and levels of performance, resulting in different personality characteristics (Smith and Ellingson, 2002). The SCSQ is developed based on the characteristics of Chinese population. The questionnaire consists of 20 items, each rated on a four-point Likert scale with scores ranging from 0 to 3, and pertains to different coping strategies for handling everyday events (Cai et al., 2021). The SSS was used to evaluate social support. A total of 10 components and 3 dimensions made up the scale (i.e., subjective support, objective support, and support utilization). Scores under 20 indicate poor social support, between 20 and 30, moderate social support, and over 30, satisfied social support (Li and Shou, 2021). The WCST is a card-matching task commonly used to evaluate cognitive flexibility and broader aspects of executive function in both research and clinical settings (Miles et al., 2021). The RBANS is a brief neurocognitive assessment tool that provides standardized measures of attention, language, memory, and visuospatial/constructional abilities (Faust et al., 2017).

### 2.5. Imaging data acquisition

Images were collected using a GE 3.0 T scanner (GE 3.0 T Signa Pioneer). The subjects were instructed to remain still, close their eyes, and stay awake. Soft earplugs and foam pads were employed to mitigate the effects of scanner noise and head movements during the imaging process. Scan parameters were repetition time/echo time = 2000/30 ms, 36 slices, 64 × 64 matrix, flip angle 90°, field of view 24 cm, slice thickness 4 mm, no gap, 250 volumes (500 s).

### 2.6. Data pre-processing

Data Processing Assistant for Resting-State fMRI (DPARSF) software was used to preprocess resting-state data in MATLAB. The slice timing and head motion in the images were corrected. To ensure the quality of the imaging data, the maximum x, y, or z displacement and angular motion for all subjects should have no more than 2 and 2°mm, respectively. The functional images were then resampled to 3 mm × 3 mm × 3 mm and normalized (Chao-Gan and Yu-Feng, 2010). An isotropic Gaussian kernel was used to smooth the processed images (fullwidth at half-maximum: 8 mm). Additionally, a linear trend removal and band-pass filtering (0.01–0.08 Hz) were processed (Song et al., 2011). Next, linear regression was used to remove a number of spurious covariates and their temporal derivatives from the data. These variables comprised the signal from a ventricular region of interest (ROI), the signal from a region centered in the white matter, and 24 head motion parameters determined *via* rigid body correction (Fox et al., 2005).

### 2.7. VMHC analysis

The REST software is used to examine VMHC (Song et al., 2011). In a brief, VMHC maps were created by computing Pearson correlations (Fisher z-transformed) between a given voxel and a mirrored voxel in the opposing hemisphere. The specifics of the VMHC analysis have been previously described in the literature (Zuo et al., 2010).

### 2.8. Statistical analysis

Data analysis in this study was conducted by using SPSS version 25.0. The chi-square test was used to examine the gender differences between the two groups. Two sample *t*-tests were employed to compare continuous variables such as age, years of education, and clinical scales. The significance level was set at *p* < 0.05.

The image data was analyzed using the DPARSF software. Two sample *t*-tests were conducted on each VMHC map to compare the groups. Then multiple comparison correction was performed based on the Gaussian random field (GRF) theory (*p* < 0.001 for voxel-level significance and *p* < 0.05 for cluster-level significance). Age, gender, education, and mean framewise displacement were used as covariates to minimize the potential influence of these variables.

For the correlation analyses, the mean VMHC values in aberrant brain areas with substantial differences between depressed patients and HCs were retrieved. The correlations between VMHC levels and clinical factors in patients with MDD were investigated using Pearson or Spearman correlation analyses. The significance level was set at *p* < 0.05 (corrected according to the Bonferroni’s correction).

### 2.9. SVM analysis

The SVM analysis was used to determine the ability of VMHC values extracted from abnormal brain regions to differentiate between HCs and patients with MDD by utilizing the LIBSVM software^1^ in MATLAB. The “leave one-out” method was used in the study.

## 3. Results

### 3.1. Demographic and clinical data

A total of 46 patients with MDD and 44 HCs were included in this study. However, due to significant head movement, the data of two HCs and four patients were excluded. As a result, the final imaging analysis included 42 patients with MDD and 42 HCs. Detailed demographic and medical information about the individuals can be found in Supplementary Table 1. The age difference between patients and HCs was significant (*p* = 0.01), and there was no significant difference between gender and years of education. There were significant differences in HAMA (*p* < 0.001), HAMD (*p* < 0.001), Neuroticism (N) (*p* < 0.001), Lie (L) (*p* < 0.001), Extraversion (E) (*p* = 0.003), SDSS (*p* < 0.001), SCSQ subscale scores (*p* < 0.001, *p* = 0.001), SSS (*p* < 0.001), NEF (*p* < 0.001), RSS (*p* = 0.025), N200 (*p* = 0.038), and P300 (*p* = 0.012) between the two groups. There was no significant difference between the two groups in SCSQ total scores, WCST, RBANS, N100, and P200.

### 3.2. VMHC analysis in depressed patients and HCs

**FIGURE 1:**
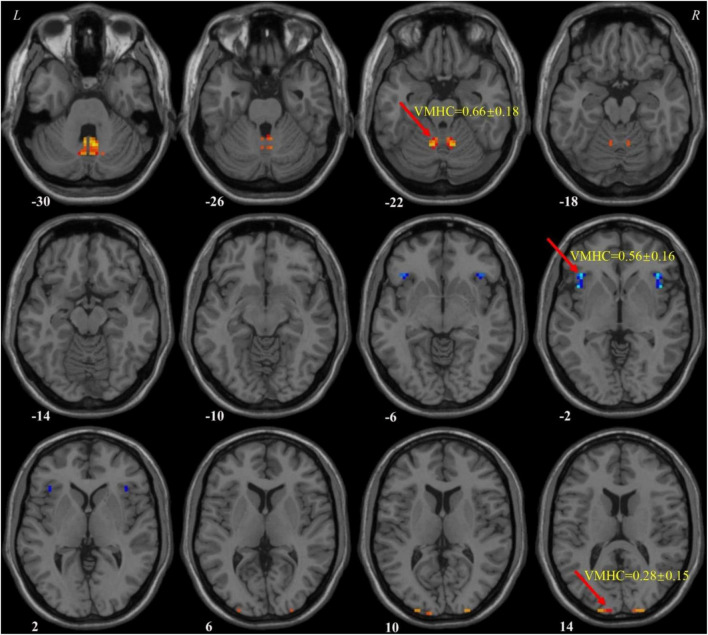
Voxel-mirrored homotopic connectivity (VMHC) values of abnormal regions in patients with MDD. MDD, major depressive disorder; VMHC, voxel-mirrored homotopic connectivity.

Our results showed that in the patients with MDD, the VMHC values of the superior/middle occipital gyrus and cerebellum 8/vermis 8/vermis 9 increased compared to HCs, while the VMHC values of the insula decreased. The specific details can be found in Table 1 and Figure 1.

**TABLE 1:** Regions with abnormal VMHC values in patients with MDD.

Cluster location	Peak (MNI)			Number of voxels	*T*-value
	**x**	**y**	**z**		
Cerebellum 8/Vermis 8/Vermis 9	±6	−57	−30	126	4.3385
Superior/middle occipital gyrus	±12	−102	12	40	4.5177
Insula	±39	18	−3	44	-2.8516

MDD, major depressive disorder; MNI, Montreal Neurological Institute; VMHC, voxel-mirrored homotopic connectivity.

### 3.3. SVM analysis

**FIGURE 2:**
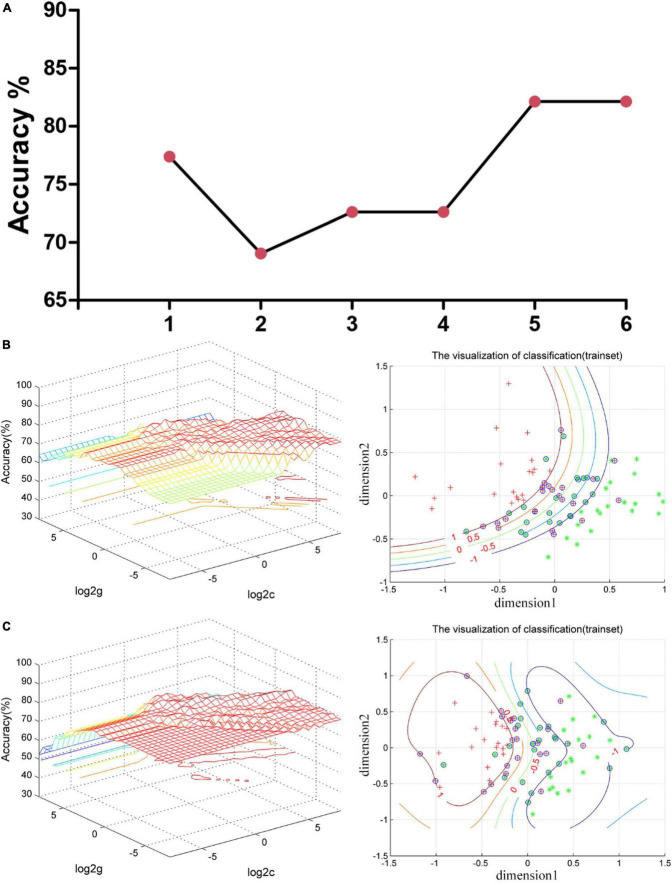
Support vector machine (SVM) results. **(A)** The accuracy of classification of six SVM analyses. One represents the VMHC values in the cerebellum 8/vermis 8/vermis 9; 2 represents the VMHC values in the superior/middle occipital gyrus; 3 represents the VMHC values in the insula; 4 represents the combination of the VMHC values in the cerebellum 8/vermis 8/vermis 9 and superior/middle occipital gyrus; 5 represents the combination of the VMHC values in the cerebellum 8/vermis 8/vermis 9 and insula; and 6 represents the combination of the VMHC values in the superior/middle occipital gyrus and insula. **(B)** SVM analysis applied the combination of the VMHC values in the cerebellum 8/vermis 8/vermis 9 and insula. Sensitivity = 80.95%, specificity = 83.33%, and accuracy = 82.14%. In the left part, the red cross represents patient with MDD, and the green asterisk represents healthy controls. The blue circle represents support vector. **(C)** SVM analysis applied the combination of the VMHC values in the superior/middle occipital gyrus and insula. Sensitivity = 78.57%, specificity = 85.71%, and accuracy = 82.14%. In the left part, the red cross represents patient with MDD, and the green asterisk represents healthy controls. The blue circle represents support vector. SVM, support vector machines; VMHC, voxel-mirrored homotopic connectivity; MDD, major depressive disorder.

According to abnormal VMHC values in various brain areas and the combination of these clusters, Figure 2A illustrates the effectiveness of differentiating patients with MDD from HCs. The sensitivity, specificity, and accuracy of the differentiation using the VMHC values in the cerebellum 8/vermis 8/vermis 9 and insula were 80.95, 83.33, and 82.14%, respectively (Figure 2B). From VMHC values in the superior/middle occipital gyrus and insula, the sensitivity was 78.57%, the specificity was 85.71%, and the accuracy was 82.14% (Figure 2C). In addition, the receiver operating curve (ROC) of applying abnormal VMHC to distinguish patients with MDD from HCs shown in Supplementary Figure 1, and the accuracy, sensitivity, and specificity of classification with ROC and SVM shown in Supplementary Table 2. ROC curve can only analyze a single brain region, while VMHC combined with SVM can analyze two brain regions. This is also an advantage of SVM, which takes into account the spatial and temporal distribution of the brain.

### 3.4. Correlation analysis result

**FIGURE 3:**
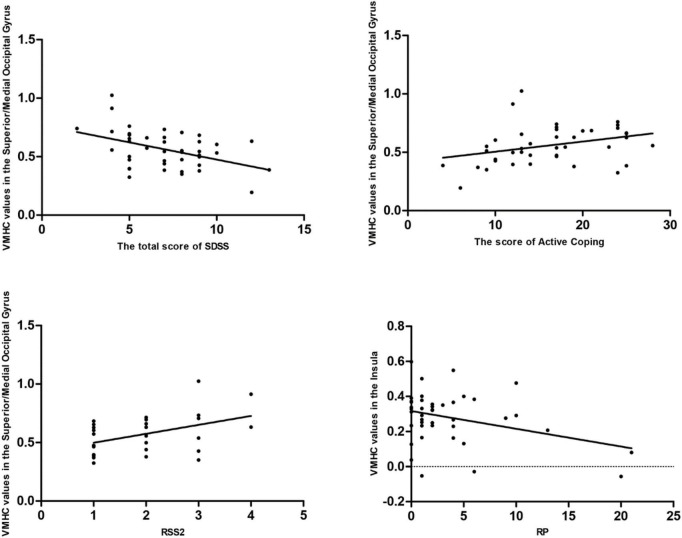
Pearson/Spearman correlations. Correlations were found as follows: (1) The VMHC values in the superior/middle occipital gyrus and the SDSS total scores (*r* = –0.441, *p* = 0.003, df = 41); (2) the VMHC values in the superior/middle occipital gyrus and the scores of Active Coping (*r* = 0.332, *p* = 0.032, df = 41); (3) the VMHC values in the superior/middle occipital gyrus and RSS2 (*r* = 0.443, *p* = 0.018, df = 27); and (4) the VMHC values in the insula and RP (*r* = –0.341, *p* = 0.027, df = 41). VMHC, voxel-mirrored homotopic connectivity; SDSS, social function defect screening scale; RSS2, responsive search score 2; RP, responses answer.

Pearson/Spearman correlation analyses revealed the following correlations: (1) the VMHC values in the superior/middle occipital gyrus and SDSS total score (*r* = −0.441, *p* = 0.003, df = 41); (2) the VMHC values in the superior/middle occipital gyrus and the scores of Active Coping (*r* = 0.332, *p* = 0.032, df = 41); (3) the VMHC values in the superior/middle occipital gyrus and RSS2 (*r* = 0.443, *p* = 0.018, df = 27); and (4) the VMHC values in the insula and RP (*r* = −0.341, *p* = 0.027, df = 41) (Figure 3).

## 4. Discussion

In this study, decreased VMHC in the insula and increased VMHC values in the sensory-motor networks may be distinctive neurobiological feature for patients with MDD, which could serve as potential imaging markers to differentiate HCs and patients with MDD. In addition, we found correlations between abnormal VMHC values and clinical/cognitive parameters in MDD. The aberrant VMHC values were further demonstrated to be promising imaging indicators of MDD by using the SVM analysis.

Event-related potential is a mature method to understand brain function in cognitive process, which is expected to make greater contribution to the identification, prediction (Tsai and Liang, 2021), treatment and prevention of mental disorders (Hajcak et al., 2019), and P300 is a typical indicator of neurophysiological ERP (Wada et al., 2019). Our research results show that there are obvious differences between patients with MDD and HCs on the cognitive level, which is consistent with a previous study (Wang et al., 2023). This may be caused by the depression of the patients and the decrease of attention to external events. The number of fixations (NEF) and response search score (RSS) in EEM are related to mental state, active attention and cognitive function (Ross et al., 2000). The neural basis of EEM has been clearly explained by a research (Lencer and Trillenberg, 2008). Studies have proved that compared with the HC group, the eye movements of patients with MDD are abnormal (Wang Y. et al., 2022). At the same time, the combination of P300 and eye movement data can improve the accuracy of auxiliary diagnosis of depression (Diao et al., 2022). ERP and EEM combined with other psychological scale data, we can see that there are obvious differences between patients with MDD and HCs.

The insula, surrounded by cortical gyrus, white matter, and basal ganglia structures (Dziedzic et al., 2022), is a center of integration for emotional, visceromotor, autonomic, and interoceptive information. Its diverse functional roles may be due to its strong connections to a broad network of cortical and subcortical regions (Wang R. et al., 2022). Studies have shown that removal of the insular lobe may disrup autonomic nerve function and alter an individual’s experience of emotions (Lacuey et al., 2019; Motomura et al., 2019). Processing input from many functioning systems involves an essential integration role for the insular cortex (Starr et al., 2009; Morita et al., 2014; Allen et al., 2016; Berret et al., 2019; Choi et al., 2022). Guo et al. (2015) found that compared with HCs, the insula of the front-limbic circuit, hate circuit, and visual regions of the patients with MDD showed decreased FC. At the same time, FC between the insula and ACC was also reduced (Krug et al., 2022). Patients with MDD showed abnormal FC between the insular subdivisions to the superior temporal sulcus, inferior prefrontal gyrus, amygdala, and posterior parietal cortex (Peng et al., 2018). Multiple studies on patients with MDD have revealed that, in comparison to HCs, their connectivity in insular lobes was reduced (Veer et al., 2010; Sliz and Hayley, 2012; Avery et al., 2014; Penner et al., 2016; Yin et al., 2018). The insula plays a key role in emotional regulation, conscious arousal, and consciousness. The decrease of insular connectivity may reflect the important regulation of negative or arousal stimuli. The decreased connectivity in the insula of patients with MDD may indicate an alteration in their perception of visceral responses and subjective sensory states (Critchley et al., 2004). Consistent with these results, we discovered that VMHC in the insula was significantly lower in patients with MDD compared to HCs, further supporting the insular crucial function in the neurological process underlying MDD. We speculate that the decrease of VMHC in the insula may be an indicator related to the emotional state of patients with MDD.

The cerebellum, located in the posterior fossa, is commonly known to control movement. However, recent studies show that it also plays a significant role in cognition and emotion (Rabellino et al., 2018; Habas, 2021; Su et al., 2021). In this study, compared with HCs, VMHC of the cerebellum 8/vermis 8/vermis 9 in patients with MDD was significantly increased. This increase in FC is often interpreted as a sign of dedifferentiation or compensatory redistribution (Cabeza et al., 2002; Grady et al., 2005; Pagen et al., 2020). Inflammatory cytokines (i.e., tumor necrosis factor and interleukin 6) can activate astrocytes and show hyperfunction (increased blood flow and metabolism) (Guo et al., 2013a). Regional hyperfunction may encourage an increase in FC and regional activity (Liu et al., 2015; Wang et al., 2016). Studies have shown that patients with MDD with MDD experience a significant increase in communication between the cerebellum and the cerebellar-anterior default mode network (Ding et al., 2022). In comparison to HCs, our previous study found a marked decrease in the FC between the cerebellum and cerebral cortex in individuals with both treatment-sensitive and treatment-resistant depression (Guo et al., 2013b). Another study showed that the cerebellar-cerebral dynamic FC of patients with MDD was lower than that of HCs (Zhu et al., 2020). This is because MDD is a multidimensional disease related to emotion, cognition, memory, etc., some special default normal functions of HCs may become dysfunctional in MDD patients, such as the VMHC of the cerebellum 8/vermis 8/vermis 9 in this study.

The findings of this study showed a significant increase in the VMHC value of the superior/medium occipital gyrus. The occipital lobe, located at the back of the cerebral hemisphere, is primarily utilized for processing visual information, and communicating with the cerebral cortex. It plays a crucial role in how facial emotions are perceived and processed (Teng et al., 2018; Li and Wang, 2021). Patients with MDD may experience aberrant cognitive processes, such as attention deficit disorder and motor delay (Yu et al., 2017). The results of this study provide additional evidence to clarify the judgment of MDD.

Support vector machine has been widely used for classify mental illnesses. The FC signal as a potential diagnostic index requires that the sensitivity or specificity of SVM be higher than 0.6 (Guo et al., 2011; Shan et al., 2021). The previous SVM analysis showed that using SVM to classify the neuroimaging biomarkers of MDD resulted in a diagnostic accuracy is 98.96% (Song et al., 2022). Compared with a study by Song et al. (2022), our study found that patients with MDD had VMHC abnormalities in extensive brain regions. At this point, the two studies are consistent. Different from Song et al. (2022) research, our research found abnormal VMHC in the insula and the sensory-motor networks. At the same time, we verify the difference between patients with MDD and HCs by measuring clinical data (such as ERP, EEM, and so on), which makes the study more convincing. The current SVM results show that the VMHC values of the insula, cerebellum 8/vermis 8/vermis 9 and superior/medium organic gyrus are greater than 0.78 in the sensitivity, accuracy, and specificity of distinguishing patients with MDD from HCs. Therefore, the VMHC values of the insula, cerebellum 8/vermis 8/vermis 9 and superior/medium organic gyrus may be used as potential imaging markers for MDD.

There are still some limitations in this study. First, the area under curve (AUC) of this study was impressive. But clearly, this is a small preliminary report that needs to be replicated. Second, we recruited patients who did not take medicine at the time of the first episode, and patients who had relapsed and did not take medicine for at least 2 weeks. The dissemination of research findings may be constrained for patients who experience relapses since the impact of antidepressant medications and number of episodes on brain spontaneous activity cannot be entirely ruled out. Finally, we only scanned the patients at baseline, so we did not know the alterations of spontaneous neuronal activity after treatment.

## 5. Conclusion

In conclusion, the study’s comparison of VMHC alterations in HCs and patients with MDD was groundbreaking. Our research results show that decreased VMHC in the insula and increased VMHC values in the sensory-motor networks may be a distinctive neurobiological feature for patients with MDD, which might be served as potential imaging markers to discriminate HCs and Patients with MDD.

## Data availability statement

The original contributions presented in this study are included in the article/Supplementary material, further inquiries can be directed to the corresponding authors.

## Ethics statement

This study was approved by the Ethics Committee of the Third People’s Hospital of Foshan (FSSY-LS202001). The patients/participants provided their written informed consent to participate in this study.

## Author contributions

CZ, HJ, and HY: writing—original draft, writing—review and editing, methodology, and software. XL, JL, QZ, WL, YO, CP, YY, and WW: validation, investigation, and resources. GX and WG: supervision, project administration, and funding acquisition. All authors contributed to the article and approved the submitted version.

## Footnotes

1
http://www.csie.ntu.edu.tw/∼cjlin/libsvm/

## References

[B1] Allen M. Fardo F. Dietz M. J. Hillebrandt H. Friston K. J. Rees G. (2016). Anterior insula coordinates hierarchical processing of tactile mismatch responses. *Neuroimage* 127 34–43. 10.1016/j.neuroimage.2015.11.030 26584870 PMC4758822

[B2] Andersson E. Crowley J. J. Lindefors N. Ljotsson B. Hedman-Lagerlof E. Boberg J. (2019). Genetics of response to cognitive behavior therapy in adults with major depression: a preliminary report. *Mol. Psychiatry* 24 484–490. 10.1038/s41380-018-0289-9 30410065 PMC6477793

[B3] Avery J. A. Drevets W. C. Moseman S. E. Bodurka J. Barcalow J. C. Simmons W. K. (2014). Major depressive disorder is associated with abnormal interoceptive activity and functional connectivity in the insula. *Biol. Psychiatry* 76 258–266. 10.1016/j.biopsych.2013.11.027 24387823 PMC4048794

[B4] Bagby R. M. Ryder A. G. Schuller D. R. Marshall M. B. (2004). The hamilton depression rating scale: has the gold standard become a lead weight? *Am. J. Psychiatry* 161 2163–2177. 10.1176/appi.ajp.161.12.2163 15569884

[B5] Berret E. Kintscher M. Palchaudhuri S. Tang W. Osypenko D. Kochubey O. (2019). Insular cortex processes aversive somatosensory information and is crucial for threat learning. *Science* 364:eaaw0474. 10.1126/science.aaw0474 31097492

[B6] Cabeza R. Anderson N. D. Locantore J. K. McIntosh A. R. (2002). Aging gracefully: compensatory brain activity in high-performing older adults. *NeuroImage* 17 1394–1402. 10.1006/nimg.2002.1280 12414279

[B7] Cai Z. Zheng S. Huang Y. Au W. W. Qiu Z. Wu K. (2021). The interactive effects of cognition on coping styles among Chinese during the COVID-19 pandemic. *Int. J. Environ. Res. Public Health* 18:3148. 10.3390/ijerph18063148 33803737 PMC8003222

[B8] Chao-Gan Y. Yu-Feng Z. (2010). DPARSF: a MATLAB toolbox for “Pipeline” data analysis of resting-state fMRI. *Front. Syst. Neurosci.* 4:13. 10.3389/fnsys.2010.00013 20577591 PMC2889691

[B9] Chen W. Hu H. Wu Q. Chen L. Zhou J. Chen H. H. (2021). Altered static and dynamic interhemispheric resting-state functional connectivity in patients with thyroid-associated ophthalmopathy. *Front. Neurosci.* 15:799916. 10.3389/fnins.2021.799916 34938158 PMC8685321

[B10] Choi S. Kim K. Kwon M. Bai S. J. Cha M. Lee B. H. (2022). Modulation of neuropathic pain by glial regulation in the insular cortex of rats. *Front. Mol. Neurosci.* 15:815945. 10.3389/fnmol.2022.815945 35493331 PMC9043281

[B11] Chu Y. Wu J. Wang D. Huang J. Li W. Zhang S. (2022). Altered voxel-mirrored homotopic connectivity in right temporal lobe epilepsy as measured using resting-state fMRI and support vector machine analyses. *Front. Psychiatry* 13:958294. 10.3389/fpsyt.2022.958294 35958657 PMC9360423

[B12] Compton R. J. Feigenson K. Widick P. (2005). Take it to the bridge: an interhemispheric processing advantage for emotional faces. *Brain Res. Cogn. Brain Res.* 24 66–72. 10.1016/j.cogbrainres.2004.12.002 15922159

[B13] CONVERGE consortium (2015). Sparse whole-genome sequencing identifies two loci for major depressive disorder. *Nature* 523 588–591. 10.1038/nature14659 26176920 PMC4522619

[B14] COVID-19 Mental Disorders Collaborators (2021). Global prevalence and burden of depressive and anxiety disorders in 204 countries and territories in 2020 due to the COVID-19 pandemic. *Lancet* 398 1700–1712. 10.1016/S0140-6736(21)02143-7 34634250 PMC8500697

[B15] Critchley H. D. Wiens S. Rotshtein P. Ohman A. Dolan R. J. (2004). Neural systems supporting interoceptive awareness. *Nat. Neurosci.* 7 189–195. 10.1038/nn1176 14730305

[B16] Dai P. Zhou X. Xiong T. Ou Y. Chen Z. Zou B. (2022). Altered effective connectivity among the cerebellum and cerebrum in patients with major depressive disorder using multisite resting-state fMRI. *Cerebellum* [Epub ahead of print]. 10.1007/s12311-022-01454-9 35933493

[B17] Demirtas M. Tornador C. Falcon C. Lopez-Sola M. Hernandez-Ribas R. Pujol J. (2016). Dynamic functional connectivity reveals altered variability in functional connectivity among patients with major depressive disorder. *Hum. Brain Mapp.* 37 2918–2930. 10.1002/hbm.23215 27120982 PMC5074271

[B18] Diao Y. Geng M. Fu Y. Wang H. Liu C. Gu J. (2022). A combination of P300 and eye movement data improves the accuracy of auxiliary diagnoses of depression. *J. Affect. Disord.* 297 386–395. 10.1016/j.jad.2021.10.028 34710500

[B19] Ding W. Cao W. Wang Y. Sun Y. Chen X. Zhou Y. (2015). Altered functional connectivity in patients with subcortical vascular cognitive impairment–A resting-state functional magnetic resonance imaging study. *PLoS One* 10:e0138180. 10.1371/journal.pone.0138180 26376180 PMC4573963

[B20] Ding Y. Ou Y. Yan H. Fu X. Yan M. Li H. (2022). Disrupted cerebellar-default mode network functional connectivity in major depressive disorder with gastrointestinal symptoms. *Front. Cell Neurosci.* 16:833592. 10.3389/fncel.2022.833592 35308120 PMC8927069

[B21] Dziedzic T. A. Bala A. Marchel A. (2022). Anatomical aspects of the insula, opercula and peri-insular white matter for a transcortical approach to insular glioma resection. *Neurosurg. Rev.* 45 793–806. 10.1007/s10143-021-01602-5 34292438 PMC8827298

[B22] Fan B. Wu P. Zhou X. Chen Z. Pang L. Shi K. (2022). Aberrant resting-state interhemispheric functional connectivity in patients with anti-N-methyl-D-aspartate receptor encephalitis. *Neuroradiology* 64 2021–2030. 10.1007/s00234-022-02983-0 35618843

[B23] Faust K. Nelson B. D. Sarapas C. Pliskin N. H. (2017). Depression and performance on the repeatable battery for the assessment of neuropsychological status. *Appl. Neuropsychol. Adult.* 24 350–356. 10.1080/23279095.2016.1185426 27267513

[B24] Fox M. D. Snyder A. Z. Vincent J. L. Corbetta M. Van Essen D. C. Raichle M. E. (2005). The human brain is intrinsically organized into dynamic, anticorrelated functional networks. *Proc. Natl. Acad. Sci. U.S.A.* 102 9673–9678. 10.1073/pnas.0504136102 15976020 PMC1157105

[B25] Fries G. R. Saldana V. A. Finnstein J. Rein T. (2022). Molecular pathways of major depressive disorder converge on the synapse. *Mol. Psychiatry* 28 284–297. 10.1038/s41380-022-01806-1 36203007 PMC9540059

[B26] Grady C. L. McIntosh A. R. Craik F. I. (2005). Task-related activity in prefrontal cortex and its relation to recognition memory performance in young and old adults. *Neuropsychologia* 43 1466–1481. 10.1016/j.neuropsychologia.2004.12.016 15989937

[B27] Guo W. Cui X. Liu F. Chen J. Xie G. Wu R. (2018). Decreased interhemispheric coordination in the posterior default-mode network and visual regions as trait alterations in first-episode, drug-naive major depressive disorder. *Brain Imaging Behav.* 12 1251–1258. 10.1007/s11682-017-9794-8 29143911

[B28] Guo W. Liu F. Liu J. Yu L. Zhang Z. Zhang J. (2013a). Is there a cerebellar compensatory effort in first-episode, treatment-naive major depressive disorder at rest? *Prog. Neuropsychopharmacol. Biol. Psychiatry* 46 13–18. 10.1016/j.pnpbp.2013.06.009 23800464

[B29] Guo W. Liu F. Xue Z. Gao K. Liu Z. Xiao C. (2013b). Abnormal resting-state cerebellar-cerebral functional connectivity in treatment-resistant depression and treatment sensitive depression. *Prog. Neuropsychopharmacol. Biol. Psychiatry* 44 51–57. 10.1016/j.pnpbp.2013.01.010 23352887

[B30] Guo W. Liu F. Xiao C. Zhang Z. Liu J. Yu M. (2015). Decreased insular connectivity in drug-naive major depressive disorder at rest. *J. Affect. Disord.* 179 31–37. 10.1016/j.jad.2015.03.028 25845747

[B31] Guo W. B. Liu F. Xue Z. M. Yu Y. Ma C. Q. Tan C. L. (2011). Abnormal neural activities in first-episode, treatment-naive, short-illness-duration, and treatment-response patients with major depressive disorder: a resting-state fMRI study. *J. Affect. Disord.* 135 326–331. 10.1016/j.jad.2011.06.048 21782246

[B32] Habas C. (2021). Functional connectivity of the cognitive cerebellum. *Front. Syst. Neurosci.* 15:642225. 10.3389/fnsys.2021.642225 33897382 PMC8060696

[B33] Hajcak G. Klawohn J. Meyer A. (2019). The utility of event-related potentials in clinical psychology. *Annu. Rev. Clin. Psychol.* 15 71–95. 10.1146/annurev-clinpsy-050718-095457 31067414

[B34] Hasin D. S. Sarvet A. L. Meyers J. L. Saha T. D. Ruan W. J. Stohl M. (2018). Epidemiology of adult DSM-5 major depressive disorder and its specifiers in the United States. *JAMA Psychiatry* 75 336–346. 10.1001/jamapsychiatry.2017.4602 29450462 PMC5875313

[B35] Hayley S. Hakim A. M. Albert P. R. (2021). Depression, dementia and immune dysregulation. *Brain* 144 746–760. 10.1093/brain/awaa405 33279966 PMC8041341

[B36] Hou Z. Song X. Jiang W. Yue Y. Yin Y. Zhang Y. (2016). Prognostic value of imbalanced interhemispheric functional coordination in early therapeutic efficacy in major depressive disorder. *Psychiatry Res. Neuroimaging* 255 1–8. 10.1016/j.pscychresns.2016.07.011 27497214

[B37] Jia C. Ou Y. Chen Y. Li P. Lv D. Yang R. (2020). Decreased resting-state interhemispheric functional connectivity in medication-free obsessive-compulsive disorder. *Front. Psychiatry* 11:559729. 10.3389/fpsyt.2020.559729 33101081 PMC7522198

[B38] Jin C. Qi S. Teng Y. Li C. Yao Y. Ruan X. (2021). Integrating structural and functional interhemispheric brain connectivity of gait freezing in Parkinson’s disease. *Front. Neurol.* 12:609866. 10.3389/fneur.2021.609866 33935931 PMC8081966

[B39] Kendall K. M. Van Assche E. Andlauer T. F. M. Choi K. W. Luykx J. J. Schulte E. C. (2021). The genetic basis of major depression. *Psychol. Med.* 51 2217–2230. 10.1017/S0033291721000441 33682643

[B40] Kozel F. A. Rao U. Lu H. Nakonezny P. A. Grannemann B. McGregor T. (2011). Functional connectivity of brain structures correlates with treatment outcome in major depressive disorder. *Front. Psychiatry* 2:7. 10.3389/fpsyt.2011.00007 21556277 PMC3089997

[B41] Krug S. Muller T. Kayali O. Leichter E. Peschel S. K. V. Jahn N. (2022). Altered functional connectivity in common resting-state networks in patients with major depressive disorder: a resting-state functional connectivity study. *J. Psychiatr Res.* 155 33–41. 10.1016/j.jpsychires.2022.07.040 35987176

[B42] Krupnik R. Yovel Y. Assaf Y. (2021). Inner hemispheric and interhemispheric connectivity balance in the human brain. *J. Neurosci.* 41 8351–8361. 10.1523/JNEUROSCI.1074-21.2021 34465598 PMC8496194

[B43] Lacuey N. Garg V. Bangert B. Hampson J. P. Miller J. Lhatoo S. (2019). Insular resection may lead to autonomic function changes. *Epilepsy Behav.* 97 260–264. 10.1016/j.yebeh.2019.04.035 31254846 PMC6916254

[B44] Lai C. H. Wu Y. T. (2014). Decreased inter-hemispheric connectivity in anterior sub-network of default mode network and cerebellum: significant findings in major depressive disorder. *Int. J. Neuropsychopharmacol.* 17 1935–1942. 10.1017/S1461145714000947 25116002

[B45] Lencer R. Trillenberg P. (2008). Neurophysiology and neuroanatomy of smooth pursuit in humans. *Brain Cogn.* 68 219–228. 10.1016/j.bandc.2008.08.013 18835076

[B46] Li N. Shou J. (2021). Risk factors for the frequent attendance of older patients at community health service centers in China: a cross-sectional study based on stratified sampling. *BMC Fam. Pract.* 22:221. 10.1186/s12875-021-01575-w 34772360 PMC8589087

[B47] Li X. Wang J. (2021). Abnormal neural activities in adults and youths with major depressive disorder during emotional processing: a meta-analysis. *Brain Imaging Behav.* 15 1134–1154. 10.1007/s11682-020-00299-2 32710330

[B48] Liu F. Zhu C. Wang Y. Guo W. Li M. Wang W. (2015). Disrupted cortical hubs in functional brain networks in social anxiety disorder. *Clin. Neurophysiol.* 126 1711–1716. 10.1016/j.clinph.2014.11.014 25534495

[B49] Liu S. Tong Y. Wang X. Yu X. Xu Y. (2022). Baseline cognitive functioning can predict the trajectory of acute treatment in first-episode major depressive disorder. *Eur. Arch. Psychiatry Clin. Neurosci.* [Epub ahead of print]. 10.1007/s00406-022-01475-9 35969275

[B50] Liu Y. Ou Y. Zhao J. Guo W. (2021). Abnormal interhemispheric homotopic functional connectivity is correlated with gastrointestinal symptoms in patients with major depressive disorder. *J. Psychiatr. Res.* 144 234–240. 10.1016/j.jpsychires.2021.10.016 34700211

[B51] Lou Y. Lei Y. Mei Y. Leppanen P. H. T. Li H. (2019). Review of abnormal self-knowledge in major depressive disorder. *Front. Psychiatry* 10:130. 10.3389/fpsyt.2019.00130 30984035 PMC6447699

[B52] Miles S. Howlett C. A. Berryman C. Nedeljkovic M. Moseley G. L. Phillipou A. (2021). Considerations for using the wisconsin card sorting test to assess cognitive flexibility. *Behav. Res. Methods* 53 2083–2091. 10.3758/s13428-021-01551-3 33754321

[B53] Morita T. Tanabe H. C. Sasaki A. T. Shimada K. Kakigi R. Sadato N. (2014). The anterior insular and anterior cingulate cortices in emotional processing for self-face recognition. *Soc. Cogn. Affect. Neurosci.* 9 570–579. 10.1093/scan/nst011 23377900 PMC4014092

[B54] Motomura K. Terasawa Y. Natsume A. Wakabayashi T. Umeda S. (2019). Anterior insular cortex stimulation and its effects on emotion recognition. *Brain Stimul.* 224 2167–2181. 10.1016/j.brs.2018.12.898 31168738

[B55] Nguyen T.-D. Harder A. Xiong Y. Kowalec K. Hägg S. Cai N. (2022). Genetic heterogeneity and subtypes of major depression. *Mol. Psychiatry* 27 1667–1675. 10.1038/s41380-021-01413-6 34997191 PMC9106834

[B56] Pagen L. H. G. van de Ven V. G. Gronenschild E. Priovoulos N. Verhey F. R. J. Jacobs H. I. L. (2020). Contributions of cerebro-cerebellar default mode connectivity patterns to memory performance in mild cognitive impairment. *J. Alzheimers Dis.* 75 633–647. 10.3233/JAD-191127 32310164 PMC7458511

[B57] Peng X. Lin P. Wu X. Gong R. Yang R. Wang J. (2018). Insular subdivisions functional connectivity dysfunction within major depressive disorder. *J. Affect. Disord.* 227 280–288. 10.1016/j.jad.2017.11.018 29128784

[B58] Penner J. Ford K. A. Taylor R. Schaefer B. Theberge J. Neufeld R. W. (2016). Medial prefrontal and anterior insular connectivity in early schizophrenia and major depressive disorder: a resting functional mri evaluation of large-scale brain network models. *Front. Hum. Neurosci.* 10:132. 10.3389/fnhum.2016.00132 27064387 PMC4811885

[B59] Rabellino D. Densmore M. Theberge J. McKinnon M. C. Lanius R. A. (2018). The cerebellum after trauma: resting-state functional connectivity of the cerebellum in posttraumatic stress disorder and its dissociative subtype. *Hum. Brain Mapp.* 39 3354–3374. 10.1002/hbm.24081 29667267 PMC6866303

[B60] Ran S. Zuo Z. Li C. Yin X. Qu W. Tang Q. (2020). Atrophic corpus callosum associated with altered functional asymmetry in major depressive disorder. *Neuropsychiatr. Dis. Treat.* 16 1473–1482. 10.2147/NDT.S245078 32606700 PMC7293967

[B61] Ross R. G. Harris J. G. Olincy A. Radant A. (2000). Eye movement task measures inhibition and spatial working memory in adults with schizophrenia, ADHD, and a normal comparison group. *Psychiatry Res.* 95 35–42. 10.1016/S0165-1781(00)00153-0 10904121

[B62] Shan X. Cui X. Liu F. Li H. Huang R. Tang Y. (2021). Shared and distinct homotopic connectivity changes in melancholic and non-melancholic depression. *J. Affect. Disord.* 287 268–275. 10.1016/j.jad.2021.03.038 33799047

[B63] Sliz D. Hayley S. (2012). Major depressive disorder and alterations in insular cortical activity: a review of current functional magnetic imaging research. *Front. Hum. Neurosci.* 6:323. 10.3389/fnhum.2012.00323 23227005 PMC3512092

[B64] Smith D. B. Ellingson J. E. (2002). Substance versus style: a new look at social desirability in motivating contexts. *J. Appl. Psychol.* 87 211–219. 10.1037/0021-9010.87.2.211 12002950

[B65] Song X. W. Dong Z. Y. Long X. Y. Li S. F. Zuo X. N. Zhu C. Z. (2011). REST: a toolkit for resting-state functional magnetic resonance imaging data processing. *PLoS One* 6:e25031. 10.1371/journal.pone.0025031 21949842 PMC3176805

[B66] Song Y. Huang C. Zhong Y. Wang X. Tao G. (2022). Abnormal reginal homogeneity in left anterior cingulum cortex and precentral gyrus as a potential neuroimaging biomarker for first-episode major depressive disorder. *Front. Psychiatry* 13:924431. 10.3389/fpsyt.2022.924431 35722559 PMC9199967

[B67] Starr C. J. Sawaki L. Wittenberg G. F. Burdette J. H. Oshiro Y. Quevedo A. S. (2009). Roles of the insular cortex in the modulation of pain: insights from brain lesions. *J. Neurosci.* 29 2684–2694. 10.1523/JNEUROSCI.5173-08.2009 19261863 PMC2748680

[B68] Steardo L. Jr. Carbone E. A. de Filippis R. Pisanu C. Segura-Garcia C. Squassina A. (2020). Application of support vector machine on fMRI data as biomarkers in schizophrenia diagnosis: a systematic review. *Front. Psychiatry* 11:588. 10.3389/fpsyt.2020.00588 32670113 PMC7326270

[B69] Su L. D. Xu F. X. Wang X. T. Cai X. Y. Shen Y. (2021). Cerebellar dysfunction, cerebro-cerebellar connectivity and autism spectrum disorders. *Neuroscience* 462 320–327. 10.1016/j.neuroscience.2020.05.028 32450293

[B70] Teng C. Zhou J. Ma H. Tan Y. Wu X. Guan C. (2018). Abnormal resting state activity of left middle occipital gyrus and its functional connectivity in female patients with major depressive disorder. *BMC Psychiatry* 18:370. 10.1186/s12888-018-1955-9 30477561 PMC6258168

[B71] Toro R. Fox P. T. Paus T. (2008). Functional coactivation map of the human brain. *Cereb. Cortex* 18 2553–2559. 10.1093/cercor/bhn014 18296434 PMC2567424

[B72] Tsai C.-C. Liang W.-K. (2021). Event-related components are structurally represented by intrinsic event-related potentials. *Sci. Rep.* 11:5670. 10.1038/s41598-021-85235-0 33707511 PMC7970958

[B73] Veer I. M. Beckmann C. F. van Tol M. J. Ferrarini L. Milles J. Veltman D. J. (2010). Whole brain resting-state analysis reveals decreased functional connectivity in major depression. *Front. Syst. Neurosci.* 4:41. 10.3389/fnsys.2010.00041 20941370 PMC2950744

[B74] Wada M. Kurose S. Miyazaki T. Nakajima S. Masuda F. Mimura Y. (2019). The P300 event-related potential in bipolar disorder: a systematic review and meta-analysis. *J. Affect. Disord.* 256 234–249. 10.1016/j.jad.2019.06.010 31200163

[B75] Wang H. Guo W. Liu F. Chen J. Wu R. Zhang Z. (2016). Clinical significance of increased cerebellar default-mode network connectivity in resting-state patients with drug-naive somatization disorder. *Medicine* 95:e4043. 10.1097/MD.0000000000004043 27428190 PMC4956784

[B76] Wang R. Mo F. Shen Y. Song Y. Cai H. Zhu J. (2022). Functional connectivity gradients of the insula to different cerebral systems. *Hum. Brain Mapp.* 44 790–800. 10.1002/hbm.26099 36206289 PMC9842882

[B77] Wang Y. Li C. Liu X. Peng D. Wu Y. Fang Y. (2023). P300 event-related potentials in patients with different subtypes of depressive disorders. *Front. Psychiatry* 13:1021365. 10.3389/fpsyt.2022.1021365 36713910 PMC9880031

[B78] Wang Y. Lyu H. L. Tian X. H. Lang B. Wang X. Y. St Clair D. (2022). The similar eye movement dysfunction between major depressive disorder, bipolar depression and bipolar mania. *World J. Biol. Psychiatry* 23 689–702. 10.1080/15622975.2022.2025616 35112653

[B79] Wei J. Wei S. Yang R. Yang L. Yin Q. Li H. (2018). Voxel-mirrored homotopic connectivity of resting-state functional magnetic resonance imaging in blepharospasm. *Front. Psychol.* 9:1620. 10.3389/fpsyg.2018.01620 30254593 PMC6141657

[B80] Wu J. Wu J. Guo R. Chu L. Li J. Zhang S. (2022). The decreased connectivity in middle temporal gyrus can be used as a potential neuroimaging biomarker for left temporal lobe epilepsy. *Front. Psychiatry* 13:972939. 10.3389/fpsyt.2022.972939 36032260 PMC9399621

[B81] Yan H. Shan X. Li H. Liu F. Guo W. (2022). Abnormal spontaneous neural activity in hippocampal-cortical system of patients with obsessive-compulsive disorder and its potential for diagnosis and prediction of early treatment response. *Front. Cell Neurosci.* 16:906534. 10.3389/fncel.2022.906534 35910254 PMC9334680

[B82] Yang J. Zheng P. Li Y. Wu J. Tan X. Zhou J. (2020). Landscapes of bacterial and metabolic signatures and their interaction in major depressive disorders. *Sci. Adv.* 6:eaba8555. 10.1126/sciadv.aba8555 33268363 PMC7710361

[B83] Yin Z. Chang M. Wei S. Jiang X. Zhou Y. Cui L. (2018). Decreased functional connectivity in insular subregions in depressive episodes of bipolar disorder and major depressive disorder. *Front. Neurosci.* 12:842. 10.3389/fnins.2018.00842 30487732 PMC6246657

[B84] Yu H. L. Liu W. B. Wang T. Huang P. Y. Jie L. Y. Sun J. Z. (2017). Difference in resting-state fractional amplitude of low-frequency fluctuation between bipolar depression and unipolar depression patients. *Eur. Rev. Med. Pharmacol. Sci.* 21 1541–1550. 28429352

[B85] Yuan K. Qin W. Liu P. Zhao L. Yu D. Zhao L. (2012). Reduced fractional anisotropy of corpus callosum modulates inter-hemispheric resting state functional connectivity in migraine patients without aura. *PLoS One* 7:e45476. 10.1371/journal.pone.0045476 23029036 PMC3454437

[B86] Zhang Q. Wu J. Pei C. Ma M. Dong Y. Gao M. (2022). Altered functional connectivity in emotional subregions of the anterior cingulate cortex in young and middle-aged patients with major depressive disorder: a resting-state fMRI study. *Biol. Psychol.* 175:108426. 10.1016/j.biopsycho.2022.108426 36152733

[B87] Zheng G. Yingli Z. Shengli C. Zhifeng Z. Bo P. Gangqiang H. (2022). Aberrant inter-hemispheric connectivity in patients with recurrent major depressive disorder: a multimodal MRI study. *Front. Neurol.* 13:852330. 10.3389/fneur.2022.852330 35463118 PMC9028762

[B88] Zhu D. M. Yang Y. Zhang Y. Wang C. Wang Y. Zhang C. (2020). Cerebellar-cerebral dynamic functional connectivity alterations in major depressive disorder. *J. Affect. Disord.* 275 319–328. 10.1016/j.jad.2020.06.062 32734925

[B89] Zuo X. N. Kelly C. Di Martino A. Mennes M. Margulies D. S. Bangaru S. (2010). Growing together and growing apart: regional and sex differences in the lifespan developmental trajectories of functional homotopy. *J. Neurosci.* 30 15034–15043. 10.1523/JNEUROSCI.2612-10.2010 21068309 PMC2997358

