# Supplementary material for: Disrupted interhemispheric coordination of sensory-motor networks and insula in major depressive disorder
Source: Front Neurosci. 2023 Mar 7;17:1135337. doi: 10.3389/fnins.2023.1135337 (PMC10028102; doi:10.3389/fnins.2023.1135337)
Supplement: Supplementary file 1 [file Data_Sheet_1.DOCX]

**Disrupted interhemispheric coordination of sensorimotor networks and insula in major depressive disorder**

**Chunguo Zhang^1^ †, Huan Jing^1^ †, Haohao Yan^2^ †, Xiaoling Li^1^, Jiaquan Liang^1^, Qinqin Zhang^1^, Wenting Liang^1^, Yangpan Ou^2^, Can Peng^1^, Yang Yu^1^, Weibin Wu^1^, Guojun Xie^1^*, Wenbin Guo^2^***

**Affiliation/address:**

^1^Department of Psychiatry, The Third People's Hospital of Foshan, Foshan, Guangdong 528000, China.

^2^Department of Psychiatry, and National Clinical Research Center for Mental Disorders, The Second Xiangya Hospital of Central South University, Changsha 410011, Hunan, China.

†: Chunguo Zhang, Huan Jing and Haohao Yan contributed equally to this work.

***Correspondence:**

Guojun Xie

Department of Psychiatry, The Third People's Hospital of Foshan, Foshan, Guangdong 528000, China.

Email: xiegjfs@126.com

Wenbin Guo

Department of Psychiatry, and National Clinical Research Center for Mental Disorders, The Second Xiangya Hospital of Central South University, Changsha 410011, Hunan, China.

Email: guowenbin76@csu.edu.cn

Table S1. Characteristics of participants.

| Variables | Patients (n = 42) | Controls (n = 42) | p-value |
| --- | --- | --- | --- |
| Age (years) | 26.43±10.79 | 35.14±12.54 | 0.001^a^ |
| Sex (male/female) | 15/27 | 18/24 | 0.503^b^ |
| Years of education (years) | 13.48±2.48 | 12.62±3.72 | 0.218^a^ |
| Framewise displacement | 0.06±0.03 | 0.07±0.03 | 0.329 ^a^ |
| HAMA | 16.60±5.70 | 2.03±2.69 | <0.001^a^ |
| HAMD | 24.80±7.22 | 2.55±3.54 | <0.001^a^ |
|  |  |  |  |
| SDSS tosal score | 7.07±2.42 | 0.02±0.15 | <0.001^a^ |
|  |  |  |  |
| EPQ |  |  |  |
| P | 51.20±8.19 | 47.23±12.68 | 0.092^a^ |
| E | 40.16±11.42 | 48.72±13.93 | 0.003^a^ |
| N | 68.37±9.19 | 45.08±10.01 | <0.001^a^ |
| L | 44.92±11.30 | 56.93±11.62 | <0.001^a^ |
|  |  |  |  |
| SCSQ |  |  |  |
| total score | 26.83±8.42 | 29.90±9.43 | 0.119^a^ |
| active coping | 16.50±6.19 | 22.90±7.35 | <0.001^a^ |
| negative coping | 10.33±4.24 | 7.00±4.35 | 0.001^a^ |
|  |  |  |  |
| SSS |  |  |  |
| total score | 28.60±8.71 | 43.14±9.33 | <0.001^a^ |
| objective support score | 7.45±3.41 | 10.93±2.85 | <0.001^a^ |
| subjective support score | 14.50±5.23 | 23.36±5.91 | <0.001^a^ |
| utilization of support | 6.64±2.12 | 8.86±2.05 | <0.001^a^ |
|  |  |  |  |
| WCST |  |  |  |
| CC | 5.00±1.21 | 5.26±1.23 | 0.328^a^ |
| RA | 46.05±2.71 | 44.00±4.02 | 0.008^a^ |
| RC | 34.55±5.58 | 34.93±3.58 | 0.711^a^ |
| RE | 11.50±6.88 | 9.02±6.21 | 0.087^a^ |
| RP | 3.57±4.94 | 2.14±3.33 | 0.124^a^ |
| RPE | 1.79±2.85 | 0.81±1.45 | 0.052^a^ |
|  |  |  |  |
| RBANS |  |  |  |
| immediate memory | 42.15±10.40 | 42.83±11.69 | 0.781^a^ |
| visuospatia/constructional | 18.73±2.16 | 17.90±2.29 | 0.100^a^ |
| language | 17.60±4.43 | 18.57±4.20 | 0.311^a^ |
| attention | 60.60±14.11 | 64.81±16.09 | 0.212^a^ |
| delayed memory | 48.33±9.65 | 49.60±10.66 | 0.574^a^ |
|  |  |  |  |
| ERP |  |  |  |
| N100 | 103.73±15.35 | 107.86±34.86 | 0.508^a^ |
| P200 | 174.53±21.59 | 177.62±23.66 | 0.545^a^ |
| N200 | 230.21±31.52 | 211.79±44.69 | 0.038^a^ |
| P300(ms) | 309.74±24.20 | 287.10±50.80 | 0.012^a^ |
|  |  |  |  |
| EEM |  |  |  |
| NEF | 21.11±5.95 | 27.52±4.32 | <0.001^a^ |
| RSS | 3.71±1.51 | 4.60±1.62 | 0.025^a^ |

HAMD = Hamilton Depression Rating Scale; HAMA = Hamilton Anxiety Rating Scale; EPQ = Eysenck Personality Questionnaire; P = Psychoticism; N = Neuroticism; E =Extraversion; L = Lie; SDSS = Social Disability Screening Schedule; SSS = Social Support Revalued Scale; SCSQ = Simplified Coping Style Questionnaire; WCST = Wsiconsin card sorting test; CC = Categories Completed; RA = Responses Answer; RC = Correct Responses; RE = Errors Responses; RP = Perseverative Responses; RPE = Perseverative Responses Errors; RBANS = Repeatable Battery for the Assessment of Neuropsyehological Status; EEM = Exploratory eye movement; NEF = number of eye fixation; RSS = responsive search score; ERP=Event related potential.

^a^ The p-values were obtained by two samples t-tests.

^b^ The p-value for sex distribution was obtained by a chi-square test.

Table S2. Accuracy, sensitivity, and specificity of classification with ROC and SVM.

|  | ROC | | | SVM | | |
| --- | --- | --- | --- | --- | --- | --- |
|  | Accuracy | sensitivity | specificity | Accuracy | sensitivity | specificity |
| Cluster 1 | 73.81% | 66.67% | 80.95% | 77.38% | 71.49% | 83.33% |
| Cluster 2 | 72.62% | 69.05% | 76.19% | 69.05% | 73.81% | 64.29% |
| Cluster 3 | 75.00% | 90.48% | 59.52% | 72.62% | 73.81% | 71.49% |


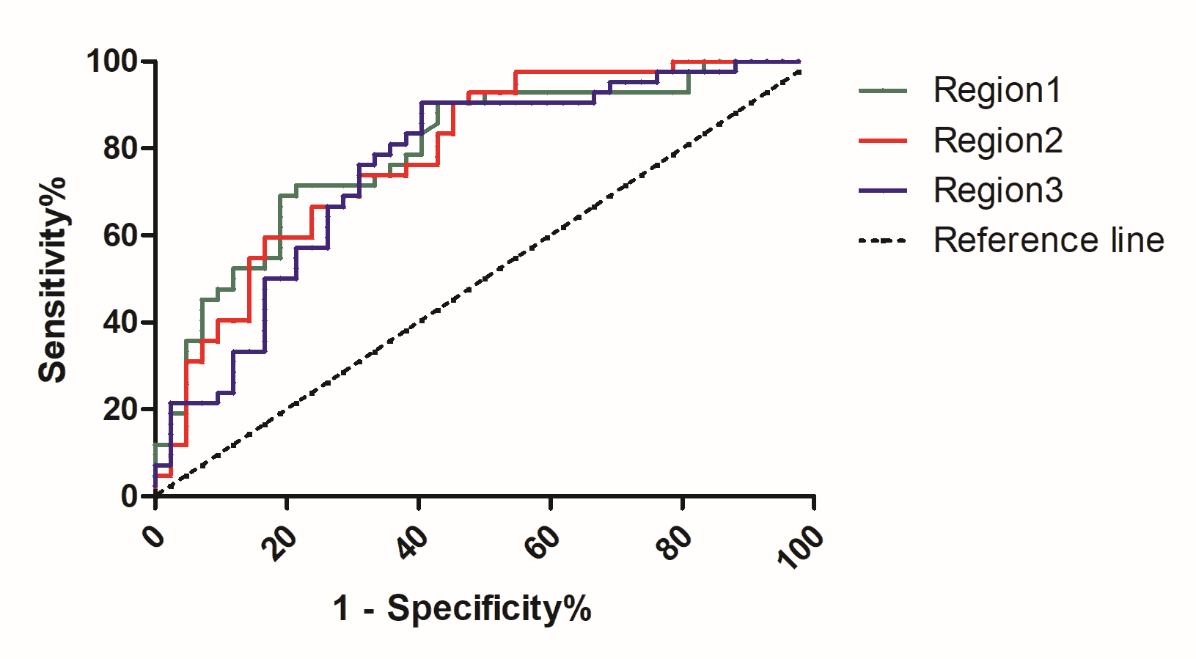


Figure S1: ROCs of applying abnormal VMHC to distinguish patients with MDD from HCs.

Region 1: Cerebellum 8/Vermis 8/Vermis 9; Region 2: Superior/Middle Occipital Gyrus; Region 3: Insula. MDD: major depressive disorder; VMHC: voxel-mirrored homotopic connectivity; ROC = receiver operating curve; HCs = Healthy controls.
